# Fusing multi-scale information in convolution network for MR image super-resolution reconstruction

**DOI:** 10.1186/s12938-018-0546-9

**Published:** 2018-08-25

**Authors:** Chang Liu, Xi Wu, Xi Yu, YuanYan Tang, Jian Zhang, JiLiu Zhou

**Affiliations:** 1 Department of Information Technology and Engineering, Chengdu University, Chengdu, 610106 China; 2 The Clinical Hospital of Chengdu Brain Science Institute, MOE Key Lab for Neuroinformation, Center for Information in Medicine, University of Electronic Science and Technology of China, Chengdu, 610054 China; 3 School of Life Science and Technology, University of Electronic Science and Technology of China, Chengdu, 610054 China; 4 Key Laboratory of Pattern Recognition and Intelligent Information Processing in Sichuan, Chengdu, 610106 China; 5 Department of Computer Science, Chengdu University of Information Technology, Chengdu, 610225 China; 6 Faculty of Science and Technology, University of Macau, Macau, China; 7 School of Physics and Electronic Engineering, Sichuan Normal University, Chengdu, China

**Keywords:** Super-resolution reconstruction, Multi-scale information fusion, Convolution network, Magnetic resonance imaging

## Abstract

**Background:**

Magnetic resonance (MR) images are usually limited by low spatial resolution, which leads to errors in post-processing procedures. Recently, learning-based super-resolution methods, such as sparse coding and super-resolution convolution neural network, have achieved promising reconstruction results in scene images. However, these methods remain insufficient for recovering detailed information from low-resolution MR images due to the limited size of training dataset.

**Methods:**

To investigate the different edge responses using different convolution kernel sizes, this study employs a multi-scale fusion convolution network (MFCN) to perform super-resolution for MRI images. Unlike traditional convolution networks that simply stack several convolution layers, the proposed network is stacked by multi-scale fusion units (MFUs). Each MFU consists of a main path and some sub-paths and finally fuses all paths within the fusion layer.

**Results:**

We discussed our experimental network parameters setting using simulated data to achieve trade-offs between the reconstruction performance and computational efficiency. We also conducted super-resolution reconstruction experiments using real datasets of MR brain images and demonstrated that the proposed MFCN has achieved a remarkable improvement in recovering detailed information from MR images and outperforms state-of-the-art methods.

**Conclusions:**

We have proposed a multi-scale fusion convolution network based on MFUs which extracts different scales features to restore the detail information. The structure of the MFU is helpful for extracting multi-scale information and making full-use of prior knowledge from a few training samples to enhance the spatial resolution.

## Background

A higher magnetic resonance image (MRI) resolution often results in fewer image artifacts, such as the partial volume effect (PVE), and a higher algorithm accuracy in the post-image processing steps (e.g., image registration and image segmentation). However, the MR resolution is affected by various physical, technological and economic limitations. Thus, increasing the spatial resolution is of considerable interest in the field of medical image processing. Conventional super-resolution (SR) methods using Bicubic and B-spline interpolation [1, 2] compute new voxel gray-values according to certain smoothness assumptions. However, these methods are not always valid in non-homogeneous areas and result in blurred images.

Super-resolution technologies have been implemented in the following two major categories. (1) During the acquisition stage, k-space data can be manipulated, and the parameters can be configured to improve the spatial resolution [3, 4]. (2) During the post-processing stage, conventional image super-resolution methods can be adapted and applied to MRI. Peled and Yeshurun [5] and Greenspan [6] applied an iterative back-projection method to 2D and 3D MRI super-resolution. The resolution enhancement [7] and non-local method [8] were also implemented and extended to reconstruct a high-resolution image from corresponding low-resolution image with inter-modality priors from another HR image [9].

Recently, sparse coding (SC)-based super-resolution approaches have been shown to have good performance and accuracy in several applications, including de-noising [10], and restoration [11]. Donoho [12] reconstructed MRI from a small subset of k-space samples to solve the super-resolution problem. Yang et al. [13] and Zeyed et al. [14] implemented sparse representations of natural images and successfully adapted these representations to MRI [15]. The sparse representation-based super-resolution method involves several steps. First, low-resolution and high-resolution dictionaries are trained by overlapping patches cropped from low- and high-resolution images, respectively. Based on this, the low-resolution images are considered sparse combinations of patches in the low-resolution dictionary space. Finally, the solved sparse coefficients are mapped onto a high-resolution dictionary space and used to reconstruct the high-resolution version. Since the conventional sparse representation method trains a dictionary based on a gradient or Sobel features, the reconstructed high-resolution images are not robust and are sensitive to noise. Additionally, the independent SC of the sequential patches cannot ensure the optimal reconstruction of entire dataset [16, 17].

Deep learning algorithms, such as the deep forward neural network or multiple layer perceptron, have recently regained their popularity [18–27] due to an improved computer infrastructure (i.e., software and hardware) and increased amount of available training data. Deep convolutional neural networks (CNN) are specialized deep forward neural networks that use a convolution operation on 1D, 2D and 3D grids (e.g., 1D time series, 2D and 3D images). Successful applications in computer vision date back several decades [28, 29]. Recently, CNN-based methods have resulted in a significantly reduced error rate that is comparable to or better than that achieved by humans in many computer vision applications, such as image classification [30], object detection [31], face recognition [32], and natural image super-resolution [26, 27]. CNN-based super-resolution learns image representations from training data similarly to all other deep learning approaches. Thus, it often produces better results than conventional feature-engineering-based methods, such as SC, when a large amount of training data is available [26], formulated the super-resolution problem into a function approximation problem. These authors have implemented a cascading convolution neural network to solve the problem of natural image super-resolution reconstruction. The end-to-end optimization of a large amount of training data produced a better result than the SC-based approach.

In the literature, a few studies have addressed the MRI super-resolution problem using the deep CNN approach. A higher dimension (3D) MRI is associated with a huge computational burden and complicates the training more than a 2D version. In addition, a large amount of training data is not always available. To overcome these challenges, we were inspired by studies using multi-scale analyses and residual networks [15, 33]; we fused multi-scale information and propagated this information along the convolution network. Unlike conventional deep CNN learning, we observed that fusing multi-scale information in a convolution network makes it easier to achieve 3D MRI super-resolution using a limited amount of training data. In addition, the experiments indicated that the multi-scale fusion convolution network (MFCN) preserved detailed image information during the reconstruction procedure, which is essential for medical image applications.

The contributions of our work include the following three aspects:

- We illustrated different convolution responses using different convolution kernel sizes experimentally and demonstrated that fusing different responses was beneficial for recovering detailed information from a low-resolution image. Conventional CNNs can learn different scale information from different convolution layers, but they are unable to integrate different scale information and decrease the error during the back-propagation procedure.
- To overcome the drawback of conventional CNNs and integrate multi-scale information induced by different convolution layers, we developed an MFCN. The proposed network, which is stacked by a multi-scale fusion unit (MFU), is a full convolution network that is capable of learning end-to-end mapping between low- and high-resolution images, makes full use of prior knowledge from high-resolution images, and uses multi-scale information to infer missed details in low-resolution images. This network exhibits an outstanding performance in MRI reconstruction. The proposed network also has a faster convergence speed than the traditional convolution network. This network is capable of learning feature maps and provides exact guidance for the design of network architecture.
- Contrary to the argument that “deeper is not better” [26], we found that a larger kernel size, an increased number of kernels, and a deeper structure are all beneficial for improving the reconstruction performance. However, these features increase the computational burden and converge more slowly. Considering the ideal trade-off between performance and speed, the adopted network structure has achieved a better performance with both simulated and real MRI data compared to some classical SR methods.

The remainder of this paper is organized as follows. "MRI super-resolution with deep learning" section presents detailed information regarding the implementation of MFCN for solving the super-resolution problem. "Experiments" section provides extensive validation using both simulated and real brain MRI datasets. A discussion and conclusion are presented in "Discussion" and "Conclusion" sections respectively.

## MRI super-resolution with deep learning

### Problem formulation

**Fig. 1:**
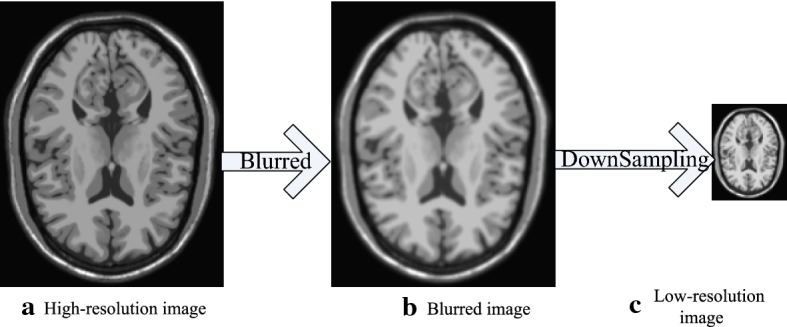
Degradation model for MRI

In the field of medical image analysis, a low-resolution MR image, *L*, can be presented as a blurred and down-sampled version of a high-resolution image, *H*, as follows:

1 $$\begin{aligned} L = DSH+e \end{aligned}$$

where *e* is the noise, *D* is a down-sampling operator, and *S* is a blurring filter. The degradation procedure is shown in Fig. 1.

In Eq. (1), the high-resolution image can be estimated by minimizing the following cost function:

2 $$\begin{aligned} \tilde{H}=\arg \mathop {\min }\limits _{} \left\| {DSH - L} \right\| ^2 \end{aligned}$$

where $\tilde{H}$ is the reconstructed high-resolution image. However, the above problem is ill-posed, and it is difficult to find a perfect solution that satisfies Eq. (2). Normally, image patches are extracted to alleviate the ill-posed nature of the problem as follows:

3 $$\begin{aligned} \tilde{H}_i =\arg \mathop {\min }\limits _{} \sum \limits _i^m {\left\| {DSH_i - L_i } \right\| ^2 } \end{aligned}$$

where $H_{i}$ and $L_{i}$ represent the *i*-th patch cropped from the high- and low-resolution images, respectively, $\tilde{H}_{i}$ is the i-th reconstructed high-resolution patch and *m* is the number of patches. Therefore, the key issue becomes identifying the mapping relationship, *DS*, in Eq. (3) that maps the high-resolution images onto the low-resolution images.

### MFCN for achieving MRI super-resolution

#### Analysis of the network architecture

**Fig. 2:**
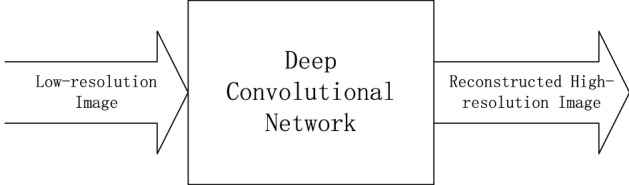
Super-resolution reconstruction based on deep convolutional network

While implementing super-resolution reconstruction using deep learning, it is natural to acquire a mapping from the low- to high-resolution images. Generally, the low-resolution image is up-sampled to have the same size as the high-resolution image before SR. Previous studies [26] have successfully implemented natural image SR with convolution neural networks. The SR based on the deep convolutional network is easy to implement due to its end-to-end learning strategy. An overview of SR based on the deep convolutional network is shown in Fig. 2.

**Fig. 3:**
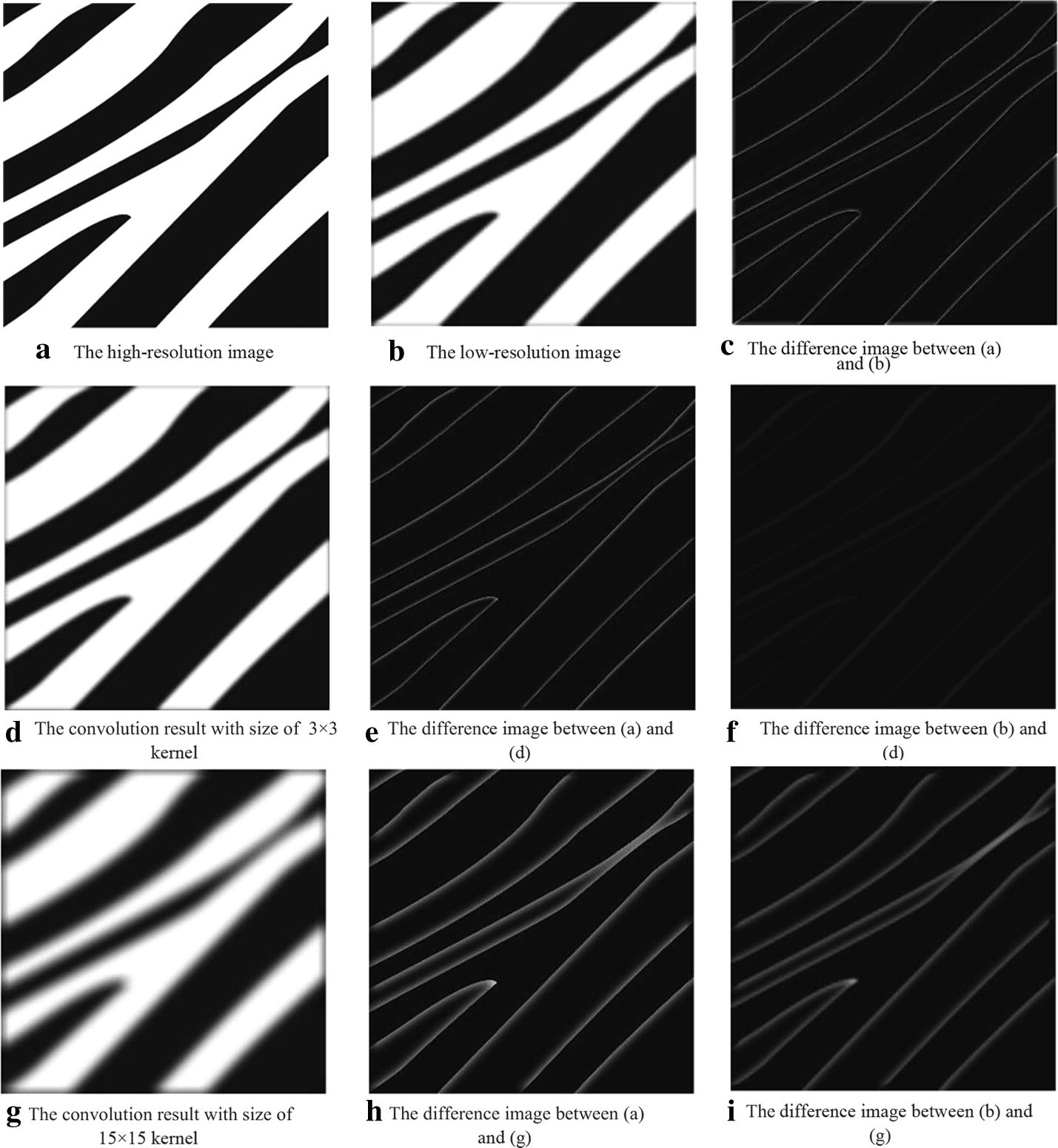
Convolution responses of convolution kernels with different sizes

The success of convolution neural networks in SR mostly depends on the contribution of the learned convolution kernels from the training samples. To investigate the effects of different convolution kernels in SR tasks, we generated two distinct kernels with sizes of $3 \times 3$ and $15 \times 15$ for a better visual representation. Then, the two kernels were applied to a simple low-resolution image. The convolution results and the difference between the high-resolution and low-resolution images are shown in Fig. 3. As shown in the first row, the main difference between the high-resolution and low-resolution images is at the edges. Therefore, the task of SR is to recover detailed information, such as edges. Furthermore, the second and third rows in Fig. 3 show that convolution operations with different kernel sizes yield varying responses along the edges, and the strengths of the responses depend on the size of the convolution kernels. Due to the receptive field range of the convolution kernels with different sizes, the larger convolution kernels induce stronger responses along the edges. Consequently, these convolution responses are extracted as multi-scale information of the convolution kernels.

#### Design of multi-scale network architecture

**Fig. 4:**
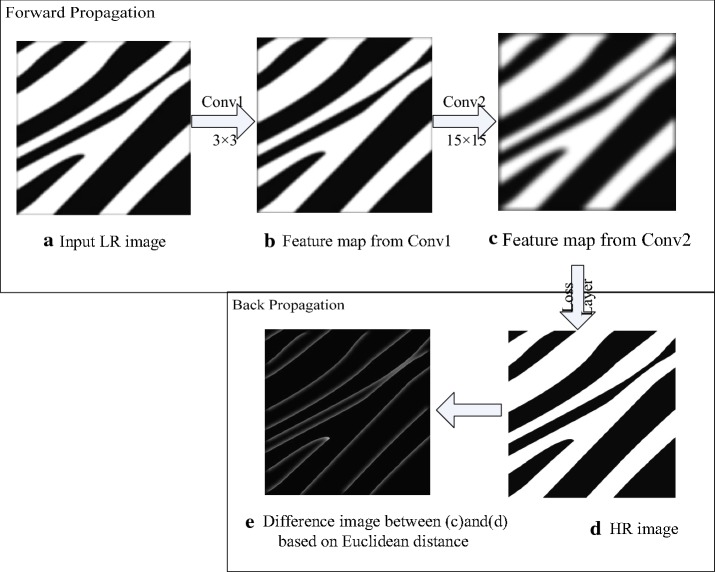
A simple convolution network for SR

Due to the forward and back propagation mechanisms in the convolution neural network, we constructed a simple convolution network stacked by two convolution layers as shown in Fig. 4. Both convolution layers have only one convolution kernel. In the convolution network, the input low-resolution images are submitted to the network and convoluted using the following convolution layers sequentially to obtain the feature maps. This procedure is called forward propagation. After the final convolution layer, the errors in the feature maps and high-resolution images, and the difference images, are computed based on the Euclidean distance of the loss layer. The difference images are very important for adjusting the kernel parameters of the final convolution layer. All parameters of each layer are adjusted using stochastic gradient descent.

**Fig. 5:**
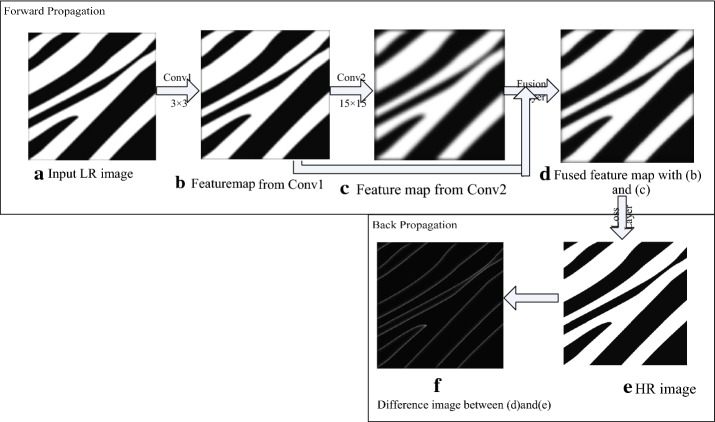
A simple MFCN for SR

Due to the multi-scale properties of different kernel sizes, fusing different scale convolution responses is assumed to accelerate the SR procedure. In the following study, we developed a simple MFCN as shown in Fig. 5. As depicted in Fig. 4, the MFCN has two convolution layers, and each layer has only one convolution kernel. We added a fusion layer to the network shown in Fig. 5. The function of the fusion layer is simply to add feature maps from (b) and (c). Initially, the fusion image had more details than the feature map in (c). Moreover, compared with the difference image I in Fig. 4, the difference image (f) in Fig. 5 is darker, which indicates less error between the recovered image and high-resolution image and is beneficial for accelerating the convergence in the training phase.

**Fig. 6:**
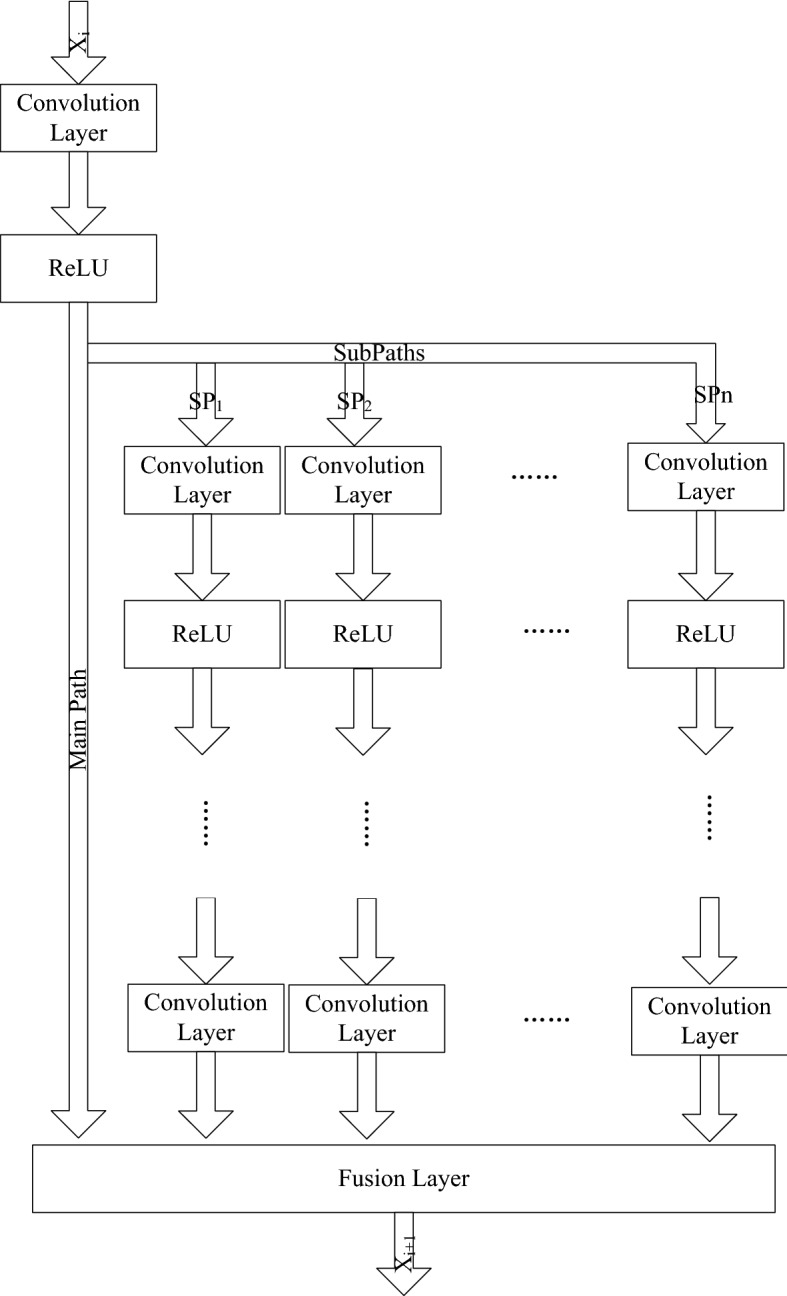
The structure of MFU

Therefore, it is desirable to design a convolution network that combines different scale information. Reconstructed images benefit from end-to-end learning of low/high-resolution images and multi-scale information propagation through the whole network structure. Inspired by residual networks [33], we defined the following structure, i.e., the MFU, to fuse different convolution paths as shown in Fig. 6:

4 $$\begin{aligned} x_{i\mathrm{{ + }}1} = f(x_i ,W_0 ) + \sum \limits _{j = 1}^J {SP_j (x_i ,\bar{W}_i )} \end{aligned}$$

where *f* represents the convolution layer and ReLU. $SP_j\, (x_i ,\bar{W}_i )$ denotes the *j*-th sub-path in which input $x_{i}$ is convoluted by some convolution kernels. *J* is the number of sub-paths. According to the main path and several sub-paths, different scale information is extracted by various convolution kernels, and then, the multi-scale information is combined in the fusion layer based on additional operations. Output $x_{i+1}$ retains more detailed information than the output from the traditional convolution network that is simply stacked by a convolution layer and helps accelerate the convergence. "Experiments" section provides a validation.

**Fig. 7:**
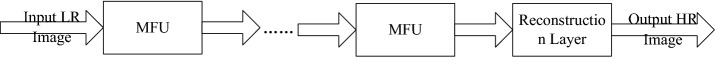
The structure of the MFCN

Based on the above-mentioned MFU, we developed the MFCN shown in Fig. 7. This network is stacked by a few MFUs and a reconstruction layer, in which the reconstruction layer is a convolution layer with one kernel.

## Experiments

To evaluate the reconstruction performance of the proposed MFCN for structural MR images, we designed an extensive set of validation experiments using both simulated and real MR images. Furthermore, several methods were employed for comparison, including bicubic interpolation, non-local mean (NLM) [11], sparse coding [13], and super-resolution convolution neural network (SRCNN) [26].

### Experimental settings

The proposed MFCN was run on an Ubuntu 14.04 with an Intel Xeon E5-2620 processor at 2.4 GHz, K80 GPU and the 96 GB of RAM based on the Caffe deep learning framework [34].

### Brain MR image sets

In this paper, the proposed MFCN was tested using different MR image sets, including both simulated and real images.

- Simulated MR images were generated using an MRI simulator and obtained from the BrainWeb brain database [35]. The simulation provides volumes acquired in the axial plane with dimensions of $181\times 217\times 181$ pixels and $1\,\text {mm}^{3}$ resolution.
- Real T1-weighted brain MR images were obtained from thirty subjects and were acquired using a GE MR750 3.0T scanner with two different spatial resolutions of $1 \,\text {mm} \times 1 \,\text {mm} \times 1 \,\text {mm}$ and $3 \,\text {mm}\times 3 \,\text {mm}\times 3 \,\text {mm}$. For the high-resolution MR images, each anatomical scan had 156 axial slices with a size of $256\times 256$ pixels. The low-resolution MR images only included 52 axial slices.

**Fig. 8:**
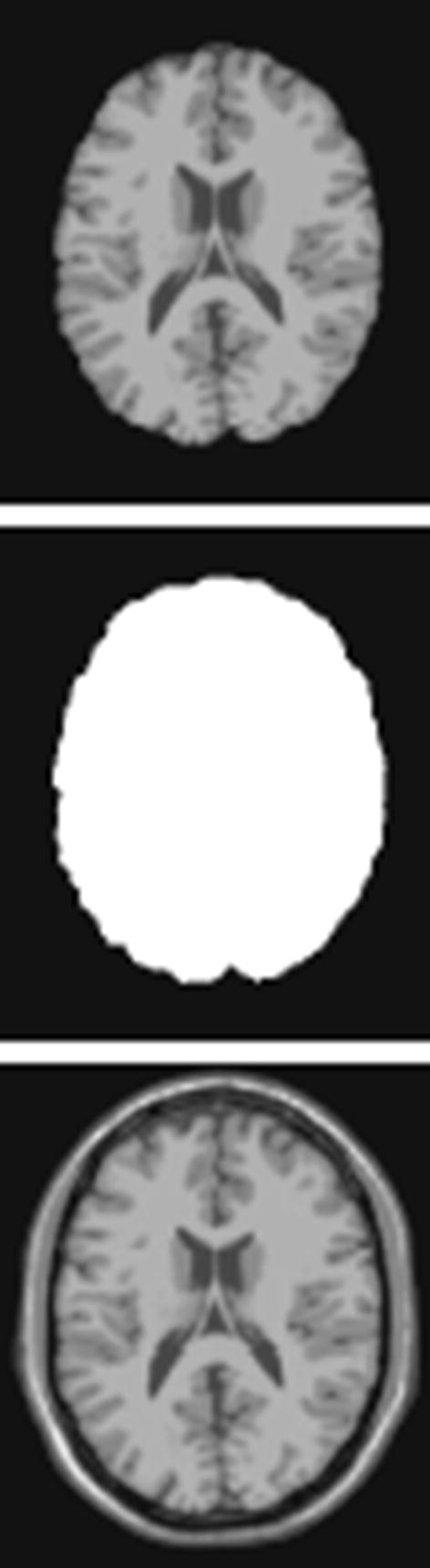
The MR image (top), its binary mask (middle) and the extracted brain region (down)

Similarly to the pre-processing step in [15], the skull and skin were removed from the MR images using a brain extraction tool (BET) [36] to eliminate the influence of the background. The resulting MR image is shown in Fig. 8. For the training set, high-resolution patches were extracted from each slice of a brain region with a size of $33 \times 33$ pixels. To obtain low-resolution patches, a blurring and down-sampling operation was applied to the extracted brain regions. Then, a bi-cubic interpolator was implemented. Finally, low-resolution image patches were acquired from the interpolated brain region.

For the learning-based method, we constructed the same training set to ensure consistency. The sparsity regularization parameter was set to 0.01 for the sparse coding-based reconstruction as reported in the literature [15]. The learning rate was set to 0.001. The network was trained using mini-batches of size 32.

### Quantitative performance measures

To quantitatively evaluate the performance of the reconstruction of different MR image sets, three different metrics were used to compare the original high-resolution images (*x*) with the reconstructed images (*y*).

- The signal-to-noise ratio (SNR) was used to compare the level of the reconstructed image with the level of the background noise:
  5 $$\begin{aligned} SNR\,(x,y) = 10\log _{10} \left( {\frac{{\sum \nolimits _k {\left| {x_k } \right| ^2 } }}{{\sum \nolimits _k {\left| {x_k - y_k } \right| ^2 } }}} \right) \end{aligned}$$
  where $x_{k}$ and $y_{k}$ are the image intensities at position *k*.
- The peak SNR (PSNR) was used to measure the reconstruction accuracy between the reconstructed image and the original image:
  6 $$\begin{aligned} PSNR\,(x,y) = 10\log _{10} \left( {\frac{R}{{\sqrt{\frac{1}{{\left| \Omega \right| }}\sum \nolimits _{k \in \Omega } {\left| {x_k - y_k } \right| ^2 } } }}} \right) \end{aligned}$$
  where $\Omega$ is the brain region, and *R* is the maximum pixel value in the low-resolution image.
- The structural similarity index (SSIM) [37] was used to measure the similarity between the two images, which is more consistent with human visual systems and perception.
  7 $$\begin{aligned} SSIM\,(x,y) = \frac{{(2\mu _x \mu _y + c_1 )(2\sigma _{xy} + c_2 )}}{{(\mu _x^2 + \mu _y^2 + c_1 )(\sigma _x^2 + \sigma _y^2 + c_2 )}} \end{aligned}$$
  where $c_{1}=(k_{1}L)^2$ , $c_{2}=(k_{2}L)^2$ , and *L* are the dynamic range, $k_{1} = 0.01$, and $k_{2} = 0.03$. The terms $\mu _x$ and $\mu _y$ are the mean values of images *x* and *y*, respectively; $\sigma _x$ and $\sigma _y$ are the standard noise variance in images *x* and *y*, respectively; and $\sigma _{xy}$ is the covariance of *x* and *y*.

### Network architecture analysis

To achieve deep learning, a large number of parameters must be tuned, which affected the reconstruction performance of the proposed network. In this section, we discuss these various factors and investigate the best trade-off between performance and speed in the simulated data. For the simulated data, the training set was constructed from the BrainWeb database using 30 real MR brain slices acquired by sampling 600 random image locations from each slice, and the test data were obtained from the slices excluded from the training set. For a baseline, the parameter configuration is listed in Tables 1,  2 and 3, where nMFU is the number of multi-scale fusion units, $MFU_{n}$ is the *n*-th multi-scale fusion units, $s_{k}$ is the size of convolution kernel, $n_{k}$ is the number of convolution kernel, nSubPath is the number of sub-paths in each MFU, and nLayer is the number of convolution layers in each sub-path.

**Table 1:** The network configurations of the baseline in MFCN

Network	nMFU	$s_{k}$/$n_{k}$ in reconstruction layer
$MFCN_{BL}$	2	5/1

**Table 2:** The $MFU_{1}$ configurations in $MFCN_{BL}$

$s_{k}$/$n_{k}$ of main path in MFU	nSubPath	nLayer in each sub-path	$s_{k}$/$n_{k}$ of in sub-path
9/32	1	1	1/32

**Table 3:** The $MFU_{2}$ configurations in $MFCN_{BL}$

$s_{k}$/$n_{k}$ of main path in MFU	nSubPath	nLayer in each sub-path	$s_{k}$/$n_{k}$ of in sub-path
3/64	1	1	1/64

#### Parameter discussion for main path

In this section, we develop several networks that have the same structure as $MFCN_{BL}$, except for a different kernel size and number in the main path in the MFU, and a final reconstruction layer to examine the reconstruction performance.

*Kernel size* Several image recognition and recognition experiments have demonstrated that if the number of kernels in each layer increases, the performance will improve. However, increasing the number of kernels also requires more time to train the network. Therefore, we compared the influence of the different kernel sizes on the reconstruction performance. The detailed parameter configuration is shown in Table 4.

**Table 4:** The different kernel size configurations

Network	$s_{k}$/$n_{k}$ in nMFU	$s_{k}$/$n_{k}$ in reconstruction layer
$MFCN_{BL}$	9/32, 3/64	5/1
$MFCN_{s_{713}}$	7/32, 1/64	3/1
$MFCN_{s_{957}}$	9/32, 5/64	7/1
$MFCN_{s_{1159}}$	11/32, 5/64	9/1

**Fig. 9:**
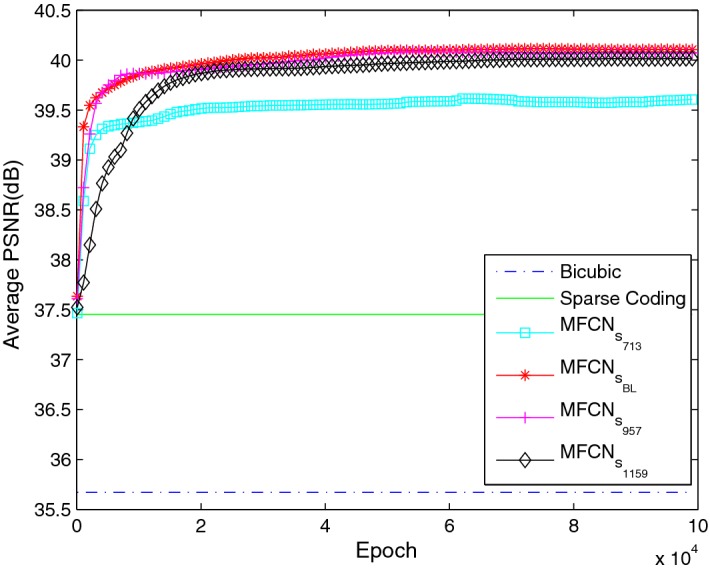
The average PSNR with different kernel sizes in the main path in MFU and the final reconstruction layer

The average PSNR with an upscaling factor of 3 is shown in Fig. 9. The proposed network with different kernel sizes always achieved a better performance than the bi-cubic interpolation and SC. Furthermore, the $MFCN_{BL}$ and $MFCN_{s_{957}}$ networks had a comparable PSNR, while the $MFCN_{s_{713}}$ network had a worse performance. One possible reason is that the $MFCN_{s_{713}}$ network had limited descriptive power for the super-resolution reconstruction due to the fewer parameters. However, we also observed that although the PSNR of the $MFCN_{s_{1159}}$ network increases as the iteration number increases, it always performs worse than the $MFCN_{BL}$ and $MFCN_{s_{957}}$ networks within limited iterative numbers, which probably illustrated that the networks with bigger kernel size need more training time to converge to achieve a better reconstruction performance, as shown in Table 5. Consequently, increasing the kernel size properly was helpful for achieving superior performance, but considering the balance between the reconstruction performance and the computational efficiency, bigger kernel size is not always good.

**Table 5:** The average SNR, PSNR, SSIM and reconstruction time of each slice with a different kernel size in the main path in MFU and the final reconstruction layer at the $10^5$ iteration

Network	SNR	PSNR	SSIM	Time (s)
$MFCN_{s_{713}}$	26.574	39.6064	0.9875	0.009123
$MFCN_{BL}$	27.0741	40.1064	0.9891	0.019142
$MFCN_{s_{957}}$	27.0415	40.0738	0.989	0.020472
$MFCN_{s_{1159}}$	26.9858	40.0181	0.9885	0.027577

*Kernel numbers* Generally, increasing the kernel number will improve performance. Based on the baseline network with 32 and 64 kernel numbers in the main path in MFU, we increased the kernel number to 64 and 96 and maintained the kernel number in the last reconstruction layer 1, called $MFCN_{n_{64961}}$. We also investigated fewer kernel numbers in the main path in MFCN with 16 and 32, referred to as $MFCN_{n_{16321}}$. The detailed configuration is shown in Table 6.

**Table 6:** The different kernel number configurations

Network	$s_{k}$/$n_{k}$ in nMFU	$s_{k}$/$n_{k}$ in reconstruction layer
$MFCN_{BL}$	9/32, 3/64	5/1
$MFCN_{n_{16321}}$	9/16, 3/32	5/1
$MFCN_{n_{64961}}$	9/64, 3/96	5/1

**Fig. 10:**
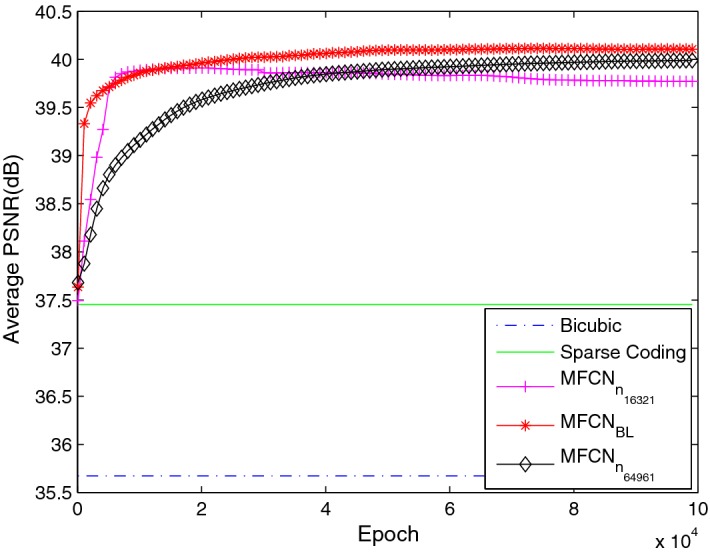
The average PSNR with different kernel numbers in the main path

These results are shown in Fig. 10. The $MFCN_{n_{16321}}$ network had the worst performance. In the initial iteration, the $MFCN_{n_{64961}}$ network had a worse performance than the baseline $MFCN_{BL}$ network. The performance of the $MFCN_{n_{64961}}$ network improved as the iteration number increased. It is possible to surpass the baseline network with additional training time likely because the $MFCN_{n_{64961}}$ network requires more learning of the network parameters. This network fails to converge during the $10^5$ epochs; therefore, it is not superior to $MFCN_{BL}$ with 32 and 64 kernel numbers.

### Sub-path parameter discussion

In this section, we discuss the influence of the sub-paths (e.g., the kernel size and the number of convolution layers in each sub-path) and the effects of preserving an ReLU layer before its addition to the MFU.

#### Kernel size of convolutional layers in the sub-paths

**Fig. 11:**
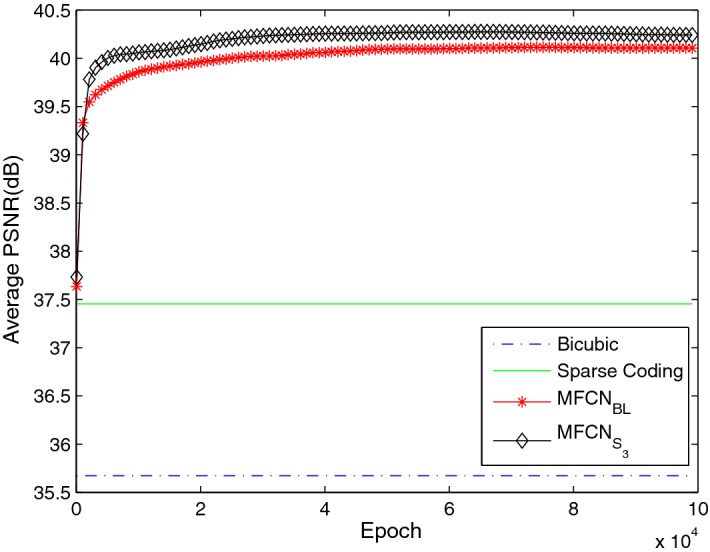
The average PSNR with different kernel sizes in the convolution layer in the sub-path in MFU

First, we discuss the kernel size of the convolution layers. As shown in Table 7, we attempted to enlarge the kernel size from $1 \times 1$ in the baseline network to $3 \times 3$ in the convolution layers in the sub-path ($MFCN_{S_{3}}$). The result is shown in Fig. 11. A superior performance was achieved in $MFCN_{S_{3}}$. As discussed in "Sub-path parameter discussion" section, the same conclusion was reached, i.e., a wider kernel size is helpful for improving performance.

**Table 7:** The different kernel number configurations

Network	$s_{k}$/$n_{k}$ of sub-path within MFU
$MFCN_{BL}$	1/32, 1/64
$MFCN_{S_{3}}$	3/32, 3/64

#### Number of convolution layers in the sub-paths

**Fig. 12:**
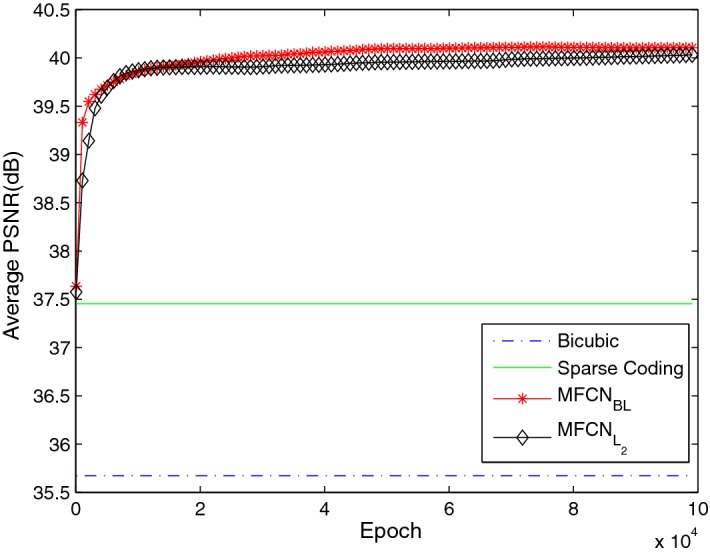
The average PSNR with different numbers of convolution layers in MFU

In the $MFCN_{BL}$, the sub-path in each MFU contains a convolution layer. Increasing the number of convolution layers in the sub-path is helpful for adding depth to the network. Therefore, we further examined networks with more convolution layers and set two convolution layers in the sub-path of each unit. The detailed configuration is shown in Table 8. Although the average PSNR shown in Fig. 12 demonstrated that the baseline network with one convolution layer ($MFCN_{BL}$) was superior to the network with two convolution layers ($MFCN_{L_{2}}$), the performance of $MFCN_{L_{2}}$ approached that of $MFCN_{BL}$ near the $10^5$ iteration and could potentially surpass the baseline networks with higher iterative numbers. $MFCN_{L_{2}}$ may need to learn more parameters and converges more slowly than $MFCN_{BL}$. Consequently, a balance between the reconstruction performance and convergence speed is needed.

**Table 8:** The different kernel number configurations

Network	nLayer in each sub-path	$s_{k}$/$n_{k}$ of sub-path within $MFU_{1}$	$s_{k}$/$n_{k}$ of sub-path within $MFU_{2}$
$MFCN_{BL}$	1	1/32	1/64
$MFCN_{L_{2}}$	2	1/32, 1/32	1/64, 1/64

#### ReLU before the fusion layer

**Fig. 13:**
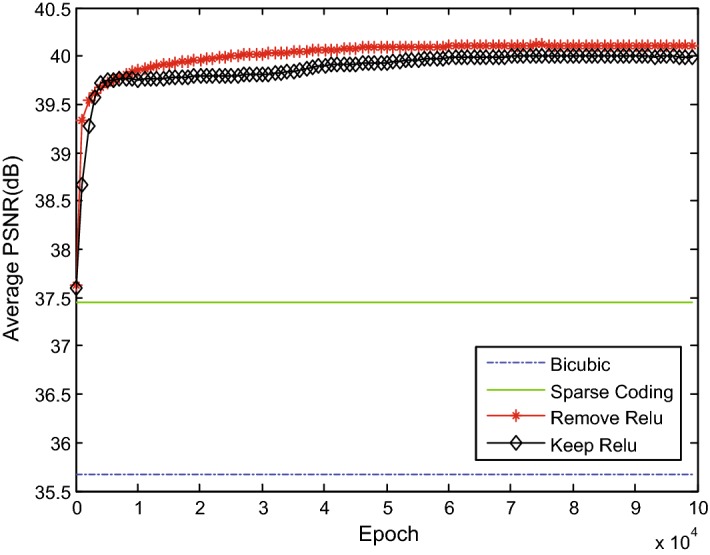
Comparison of MFU with and without ReLU before adding the fusion layer

Previous studies [33] have shown that the “residual” unit should be in the range of $\left( { - \infty , + \infty } \right)$ and suggested removing the ReLU before the addition of the fusion layer to achieve a lower error in the image classification. To confirm the performance of ReLU in MFU for MRI super-resolution reconstruction, we also investigated a network structure by adding ReLU before the addition of each MFU. The other settings remained the same as those in the baseline network ($MFCN_{BL}$). As shown in Fig. 13, removing ReLU before adding the fusion layer exceeded the performance compared to when ReLU was maintained.

### Number of MFUs

**Fig. 14:**
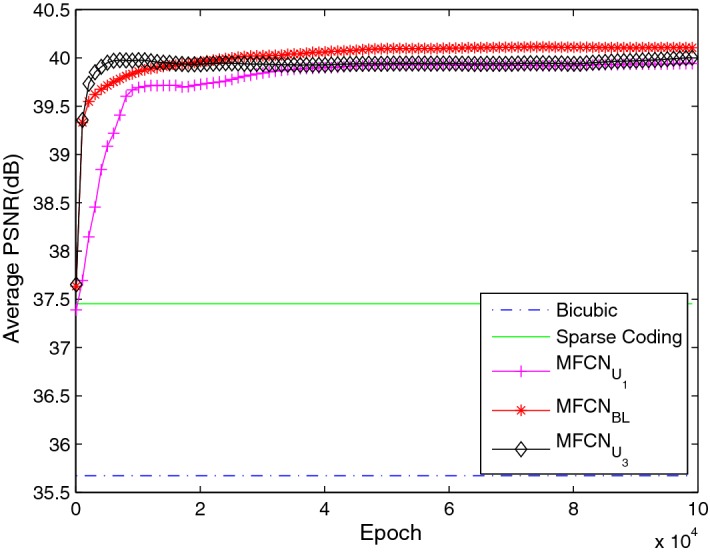
The average PSNR with different numbers of MFUs

Several deep learning image recognition and classification experiments have demonstrated that performance can benefit from increasing the network depth. However, previous studies [26] have claimed that deeper networks do not always achieve an improved performance. In addition to increasing the number of convolution layers in the sub-path in "Sub-path parameter discussion", we attempted to deepen the network by adding several MFUs. The detailed configuration is shown in Table 9. As shown in Fig. 14, a network with one MFU ($MFCU_{U_{1}}$) had a worse performance than the baseline network with two MFUs ($MFCN_{BL}$). Initially, the networks with three MFUs ($MFCU_{U_{3}}$) were superior to the baseline network, but their performance worsened after approximately 20K iterations, and the curve increased by nearly $10^5$ iterative numbers. Therefore, it is difficult to achieve the same conclusion as [26]. We believe that this trend does not oppose the advantage of the network depth. Deeper networks cannot converge within $10^5$ iterations due to the requirement of more learned parameters, which leads to a worse performance than that of the baseline network from $2 \times 10^4$ to $10^5$ iterations.

**Table 9:** The different MFUs configurations

Network	$s_{k}$/$n_{k}$ of sub-path within $MFU_{1}$	$s_{k}$/$n_{k}$ of sub-path within $MFU_{2}$	$s_{k}$/$n_{k}$ of sub-path within $MFU_{3}$
$MFCN_{BL}$	1/32	1/64	N/A
$MFCU_{U_{1}}$	1/32	N/A	N/A
$MFCU_{U_{3}}$	1/32	1/32	1/64

### Learned feature maps

**Fig. 15:**
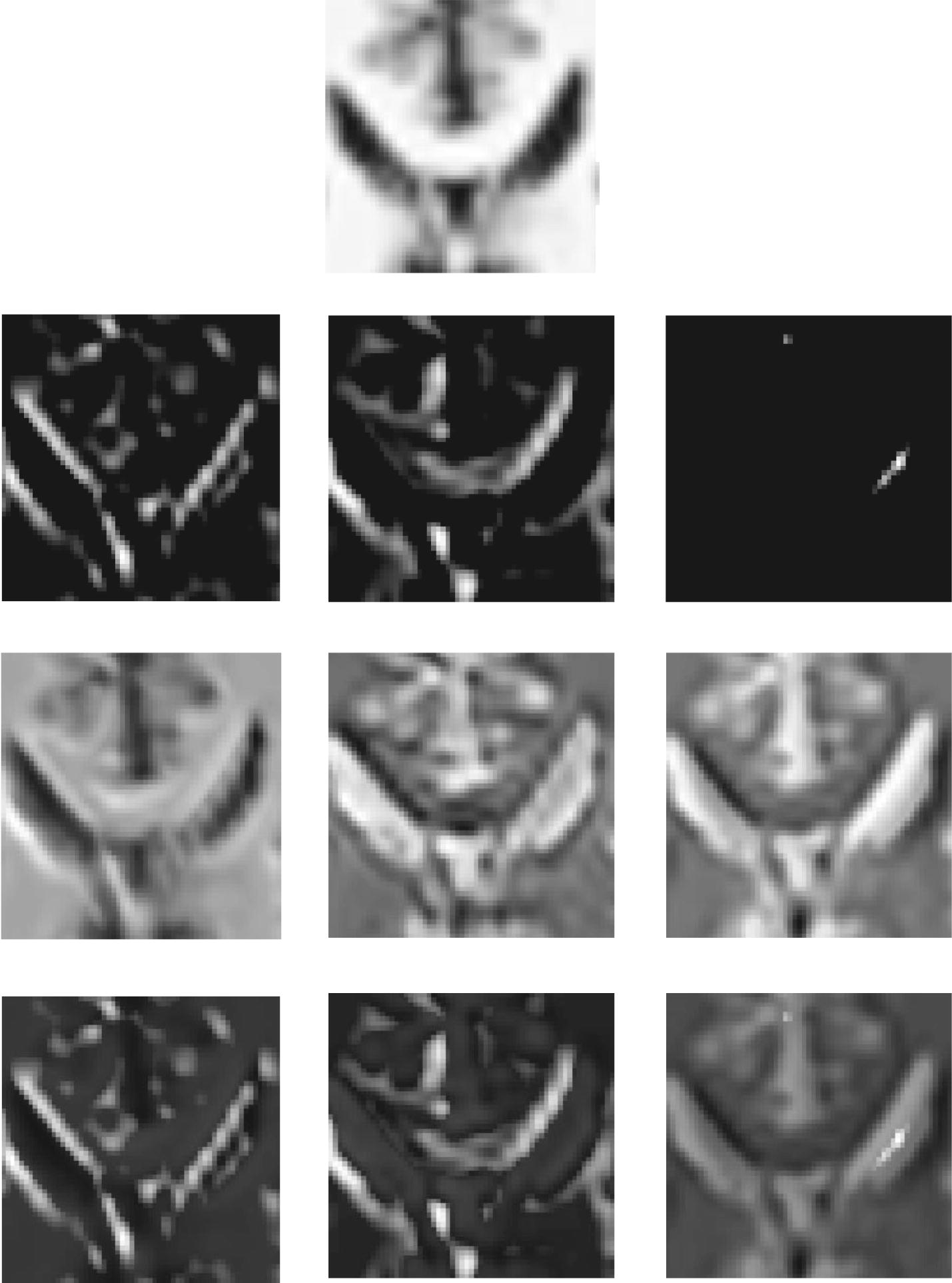
Low-resolution image (first row), feature maps learned by the main path (second row), feature maps learned by a sub-path in MFU (third row) and feature maps after the addition to an MFU (fourth row)

**Fig. 16:**
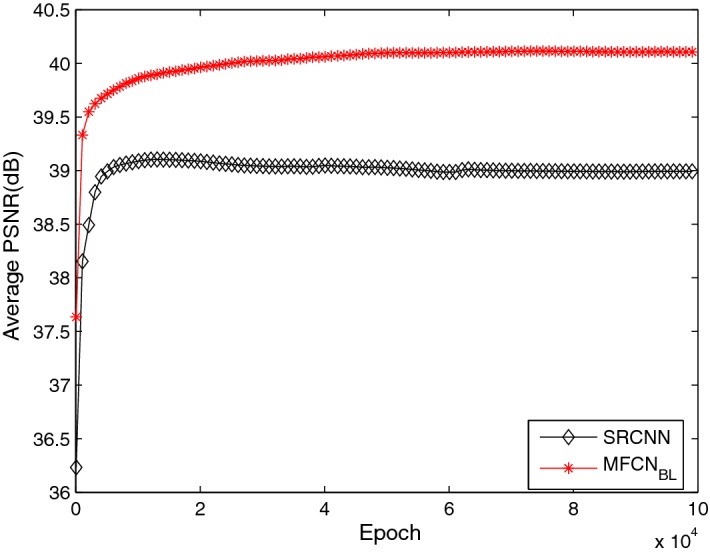
Comparison of SRCNN and MFCN

To investigate why the proposed network is capable of super-resolution reconstructions, some feature maps were studied using different layers and are shown in Fig. 15. As shown in Fig. 15, different kernels in the main path extract distinct information from low-resolution images, such as different directions, as shown in the second row. Convolution layers in the sub-path recover different modalities based on the feature maps in the second row as shown in the third row. The feature maps in the second and third rows are complementary, and the final fusion layer with the addition operation in MFU is helpful for combining the complementary information as shown in the fourth row. For a better understanding of MFU, we further compared $MFCN_{BL}$ and the traditional convolution network (SRCNN) using the same configurations. The results are shown in Fig. 16. The MFCN was always superior to the SRCNN.

In summary, we investigated the parameter settings of the proposed network and decomposed MFU to visualize the feature maps of the main path and sub-path. Each of these experiments indicated that a larger kernel size, an increased kernel number in the convolution layers, and a deeper network are helpful for improving the reconstruction performance. However, many parameters need to be learned, and the convergence is, therefore, slow. Consequently, we must compromise between performance and efficiency.

### Comparisons to state-of-the-art approaches

In previous experiments, the influence of different parameters on networks and reconstruction performance has been discussed. To balance the performance and computational efficiency, we adopted the above-mentioned baseline network due to its good performance-speed trade-off. Once the network architecture was fixed, the super-resolution reconstruction experiments were carried out to validate the performance of the proposed method. In this section, quantitative and qualitative results of the proposed method were compared with results of certain classical methods for different up-sampling factors *f*, including *f* = 2, 3 and 4. The implementation of existing methods was achieved using publicly available codes provided by the authors. For the MFCN and SRCNN, we trained the network using $10^5$ iterations.

#### Different up-sampling factors

**Fig. 17:**
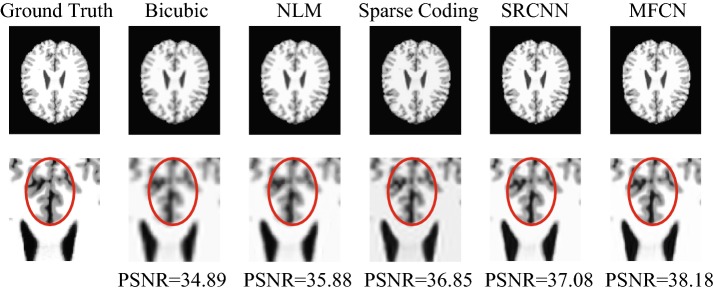
Visual comparison of different methods using a BrainWeb Dataset

As shown in Table 10, the proposed method always yielded the best scores with different evaluation metrics. Figure 17 also illustrates the reconstructed MR images using different methods in a single slice. Notably, within the red circle of Fig. 17, it can be found that the reconstructions based on MFCN were able to restore more detailed information for the MR images than those based on the other classical methods.

**Table 10:** Quantitative evaluation (RMSE, SNR, PSNR, and SSIM) of different up-sampling factors using BrainWeb MR images

Eval. met	Scale	Bicubic	NLM	Sparse coding	SRCNN	MFCN
RMSE	2	2.5077	2.1112	1.7747	1.4836	1.2026
	3	4.2038	3.7707	3.4262	2.8937	2.5415
	4	5.849	5.5588	5.0577	4.6946	4.5196
SNR	2	27.1376	28.636	30.1495	31.7244	33.5375
	3	22.6394	23.5894	24.422	25.9595	27.0741
	4	19.767	20.2123	21.0326	21.7213	22.0571
PSNR	2	40.1699	41.6684	43.1819	44.7567	46.5698
	3	35.6717	36.6218	37.4262	38.9918	40.1064
	4	32.7993	33.2446	34.056	34.7536	35.0895
SSIM	2	0.9891	0.9923	0.9945	0.9963	0.9975
	3	0.9678	0.9743	0.9788	0.9864	0.9891
	4	0.9375	0.9434	0.9529	0.9639	0.9662

#### Evaluation of real data

**Fig. 18:**
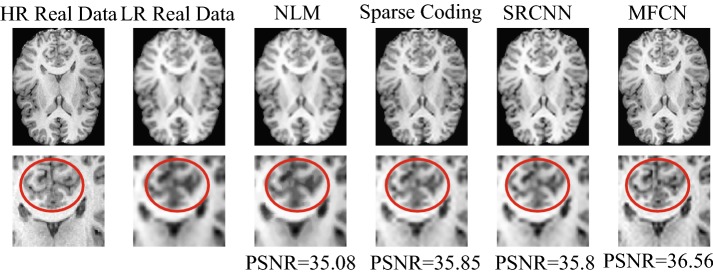
Visual comparison of the different methods using real data

**Fig. 19:**
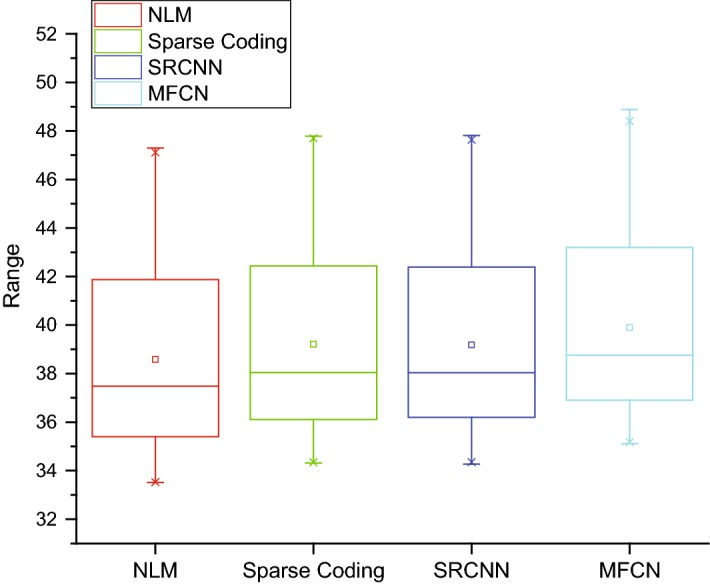
Boxplot of PSNR using different methods with a real dataset

We further examined the performance of the MFCN using a real dataset. We selected fifteen subjects as the training data and the remaining subjects as the test data. Figure 18 shows representative image reconstruction results using various methods. From left to right, the first row shows the high-resolution image, the corresponding low-resolution image, and the results of NLM, sparse coding, SRCNN, and MFCN. The close-up views of the selected regions are also shown for better visualization. The results of NLM show severe blurring artifacts, and the results of sparse coding are better than those of NLM. The contrast is enhanced in the SRCNN results, while the proposed MFCN is the best for preserving edges and achieving the highest PSNR value as shown in Fig. 18. The quantitative results using the real datasets are illustrated in Fig. 19. As shown in Fig. 19, the total distribution of PSNRs for MFCN are better than others; The mean (small square in the box) and the median (the horizontal line in the box) of PSNR for MFCN are also greater than other one. Therefore, the proposed method significantly outperformed all compared methods.

## Discussion

It is well known that the convolution neural network has a large number of network parameters and is needed for training with a large dataset to avoid over-fitting. However, due to limited MRI training data, it is difficult to achieve superior reconstruction using a standard convolution network. In this work, we developed an MFCN for MRI super-resolution reconstruction and achieving end-to-end (one-to-one) mapping between low and high-resolution images. Instead of a traditional convolution network, the network is stacked by MFUs. Each MFU consists of a main path and several sub-paths, and all paths are finally added to the fusion layer to fuse multi-scale information. We conducted several experiments and demonstrated that when the training data are limited, the proposed network always achieves superior reconstruction results using both simulated and real data compared with traditional SR methods, such as bi-cubic, NLM, sparse coding, and SRCNN.

An additional concern is the slow convergence speed caused by the traditional convolution network structure. Regarding fusing multi-scale information from the main path and sub-paths in MFU, we found that the proposed network achieves faster convergence speed than the traditional convolution network SRCNN. As shown in Fig. 16, with the same parameter settings, the proposed network converges after 3000 epochs while SRCNN converges after 5000 epochs. Furthermore, the proposed network achieves a higher PSNR value in the same epoch. Moreover, the proposed network can recover more detailed information and has better visual effects as shown in Figs. 17 and 18.

Finally, currently used convolution networks for image super-resolution usually extract detailed information on a single scale, and the back propagation process fails to utilize prior knowledge of the high-resolution images.

According to previous research on SR [38], the extraction of multi-scale information improves the reconstruction results. Using the proposed convolution network, we also experimentally validated that differently sized convolution kernels can acquire multi-scale information as shown in Fig. 3. We found that multi-scale information can be merged and transmitted from one MFU to the next as shown in Fig. 15. Thus, the proposed network can recover detailed information and achieve better reconstruction performance.

Our results are inconsistent with the conclusion reached using SRCNN [26] that “deeper is not better”, and many experiments investigating parameter settings have illustrated that incremental network depths and kernel sizes are helpful for improving the reconstruction results. Generally, we should seek a balance between computational efficiency and reconstruction performance. Using both simulated and real data, the proposed network has demonstrated visually and quantitatively prominent performance for MRI super-resolution reconstruction.

## Conclusion

In this paper, we demonstrated an MFCN for MRI super-resolution. The network is able to learn end-to-end mapping from low/high-resolution images. Simultaneously, due to the fusion of different paths in MFU, the network can extract multi-scale information to recover detailed information and accelerate the convergence speed. The extensive experiments using simulated and real data have also demonstrated that this approach is superior to other traditional methods. In addition, the proposed network architecture and experimental framework can be applied to other medical super-resolution reconstructions, such as in CT and diffusion-weighted MR imaging.
